# The progress of miR-205 regulating apoptosis in cancer

**DOI:** 10.3389/fonc.2025.1532659

**Published:** 2025-09-10

**Authors:** Dandan Chai, Hua Du, Yingxu Shi

**Affiliations:** ^1^ Department of Laboratory, Affiliated Hospital of Inner Mongolia Medical University, Hohhot, China; ^2^ Department of Pathology, Basic Medical College, Inner Mongolia Medical University, Hohhot, Inner Mongolia, China; ^3^ Department of Pathology, Affiliated Hospital of Inner Mongolia Medical University, Hohhot, Inner Mongolia, China

**Keywords:** apoptosis, cancer, microRNA, miR-205, programmed cell death

## Abstract

MicroRNAs (miRNAs) constitute a class of small non-coding RNAs that play a pivotal role in post-transcriptional gene regulation. The dysregulation of miRNAs has been widely implicated in the pathogenesis of diverse human cancers. Among these, miR-205 has attracted considerable attention owing to its aberrant expression patterns in multiple cancer types, where it regulates tumor initiation and progression via diverse molecular mechanisms. Apoptosis, a fundamental biological process essential for cellular homeostasis, represents a tightly regulated form of programmed cell death that significantly influences cancer development under both physiological and pathological conditions. In malignant cells, miR-205 exhibits a dual regulatory role by modulating apoptosis-related signaling pathways and their downstream target genes, thereby displaying both oncogenic and tumor-suppressive functions. This comprehensive review systematically explores recent advances in understanding the functional role of miR-205 in apoptosis regulation across a spectrum of human malignancies and highlights its potential therapeutic implications for future cancer therapies.

## 1 Introduction

The global burden of cancer continues to escalate, with both incidence and mortality rates demonstrating a persistent upward trajectory, underscoring the profound and growing impact of neoplastic diseases on public health systems worldwide (1), A pivotal determinant underlying this concerning phenomenon is the ability of malignant cells to evade programmed cell death through diverse molecular mechanisms, thereby facilitating tumor progression and therapeutic resistance (2). Apoptosis, a genetically programmed form of cell death, constitutes a critical biological mechanism that suppresses the proliferation of damaged or aberrant cells. The evasion of this regulatory process by malignant cells represents a fundamental hallmark of cancer, contributing to tumor progression and conferring resistance to therapeutic interventions. Within this context, there is increasing recognition that therapeutic strategies targeting the induction of programmed cell death through precise modulation of apoptotic pathways may constitute a promising and potentially transformative approach in oncology. However, despite this therapeutic potential, the current understanding of the molecular mechanisms underlying apoptotic regulation in neoplastic cells remains fragmentary and incomplete, necessitating further comprehensive investigation (3). The ultimate objectives are to enhance overall survival rates and improve the quality of life for cancer patients. Extensive research efforts have been devoted to elucidating the regulatory relationships between microRNAs (miRNAs) and apoptotic pathways in neoplastic cells. miRNAs represent a class of small, evolutionarily conserved non-coding RNA molecules, typically comprising 18 to 24 nucleotides in length. These regulatory molecules exert their biological functions through sequence-specific interactions with the 3’-untranslated regions (3’-UTRs) of target messenger RNAs (mRNAs), thereby modulating post-transcriptional gene expression (4, 5). A significant proportion of miRNAs are fundamentally implicated in both genetic and epigenetic alterations that influence cancer-associated gene networks. Dysregulation of miRNA expression profiles can lead to developmental perturbations and has been demonstrated to play a pivotal role in oncogenic transformation and tumorigenic processes (6). MicroRNAs exert their regulatory influence on oncogene function across multiple pivotal stages of tumorigenesis, encompassing tumor initiation, progression, and metastatic dissemination, through precise targeting and modulation of cancer-associated genes (7). In this comprehensive review, we systematically examine the functional roles and molecular mechanisms through which miR-205 regulates apoptotic processes in malignant cells across diverse cancer types (8). Furthermore, we critically evaluate its clinical significance and potential therapeutic value in tumor regulation, while establishing a theoretical framework to facilitate its translation into clinical applications for cancer prevention and treatment strategies.

## 2 Overview of miR-205

miR-205 was initially characterized through comparative genomic analyses of murine and Fugu rubripes sequences. Subsequent investigations have further identified its conserved expression patterns in zebrafish (Danio rerio) and human genomes, demonstrating its evolutionary significance across vertebrate species (9–11). miR-205 is genomically located at chromosome 1 (1q32.2) in humans. This microRNA is characterized by a highly conserved core sequence (5’-UCCUUUCAUUCCACCGGAGUCUG-3’) that is essential for its functional activity and specific molecular interactions with target mRNAs (12).

### 2.1 Physiological function of miR-205

**Figure 1:**
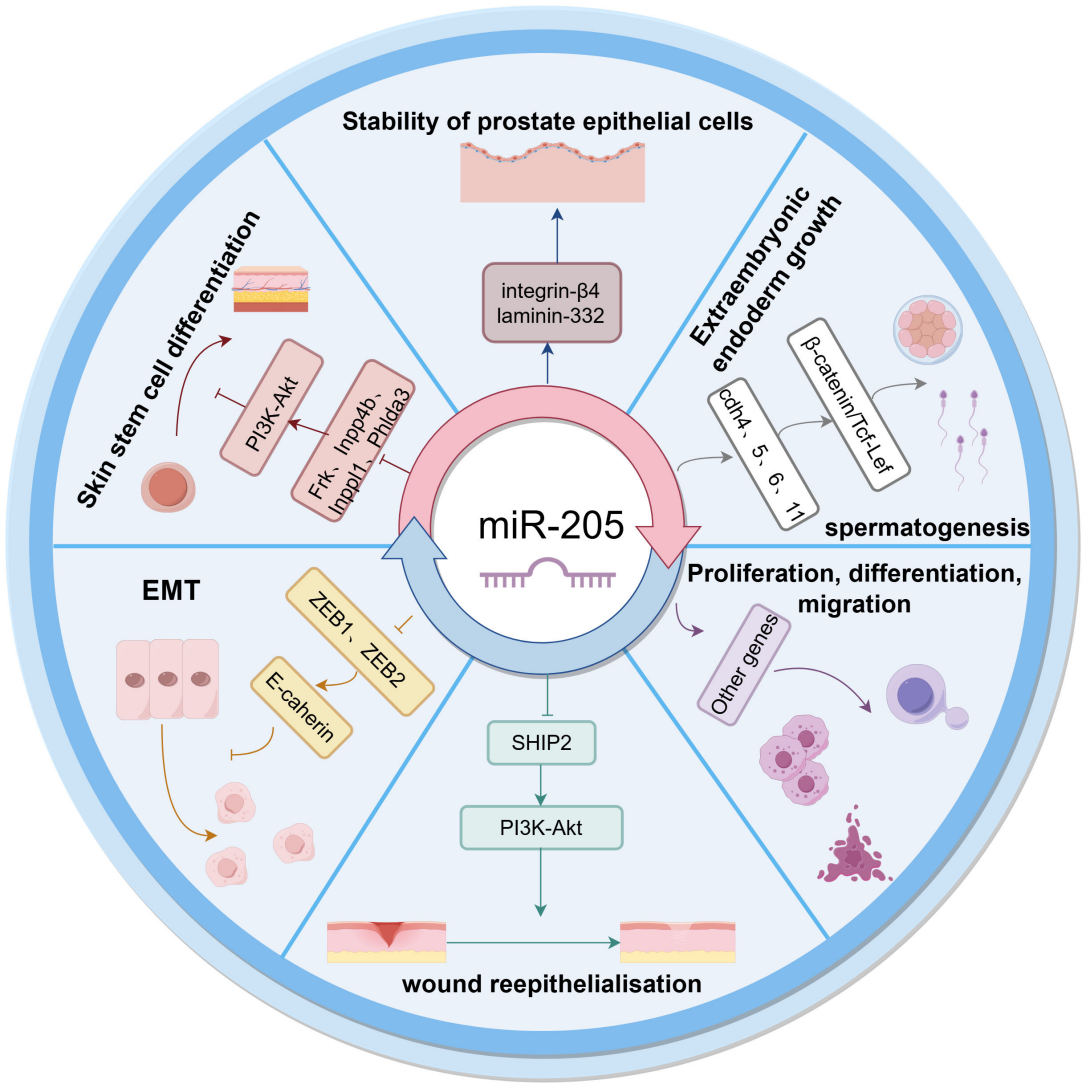
Physiological role of miR-205.

miR-205 has been demonstrated to play a critical role in numerous essential physiological processes (**Figure 1**). Specifically, it regulates skin stem cell differentiation, is indispensable for epithelial cell homeostasis, inhibits epithelial-mesenchymal transition (EMT), and modulates cellular proliferation and differentiation processes. Wang et al. demonstrated that in miR-205 knockout skin stem cells, the expression levels of negative regulators of the PI3K-AKT pathway, including Frk, Inpp4b, Inppl1, and Phlda3, were significantly upregulated. This led to the inhibition of PI3K signaling and a marked downregulation of phosphorylated AKT (p-AKT) levels, resulting in the premature termination of skin stem cell differentiation (13). Furthermore, miR-205 has been shown to facilitate cutaneous wound healing through the promotion of keratinocyte migration, thereby enhancing the re-epithelialization process (14). Yu et al. further demonstrated that elevated expression of miR-205 results in the downregulation of lipid phosphatase SHIP2, consequently activating the PI3K-Akt signaling pathway in keratinocytes. This pathway activation is essential for inhibiting apoptotic processes and plays a critical role in promoting efficient wound healing (15). Basal cells are recognized as progenitor cells responsible for epithelial cell generation. miR-205 exhibits high expression levels in prostate basal cells, where it mediates the deposition of laminin-332 and its cognate receptor integrin-β4 within the basement membrane. This molecular mechanism is crucial for maintaining prostate basal cell homeostasis and preserving the structural integrity and physiological function of the prostate gland (16). The investigation conducted by Teta et al. has revealed that miR-205 exhibits prominent expression in epidermal keratinocytes while being conspicuously absent in follicular cells, demonstrating its tissue-specific expression pattern in epithelial compartments (17). E-cadherin, a critical transmembrane protein essential for maintaining intercellular adhesion in epithelial tissues, is negatively regulated by the transcriptional repressors ZEB1 and ZEB2. Emerging evidence demonstrates that miR-205 directly targets and downregulates ZEB1 and ZEB2, thereby upregulating E-cadherin expression and effectively inhibiting epithelial-mesenchymal transition (EMT) in epithelial cells. These findings underscore the pivotal role of miR-205 in modulating E-cadherin-mediated cell adhesion and maintaining epithelial phenotype integrity (18, 19). Furthermore, miR-205 has been demonstrated to play a significant regulatory role in embryonic developmental processes. Elevated expression levels of miR-205 have been shown to induce the upregulation of multiple members of the calmodulin family (including Cdh4, Cdh5, Cdh6, and Cdh11) as well as various genes associated with cell adhesion. This upregulation subsequently activates the β-catenin/Tcf-Lef signaling pathway, which is critically involved in extraembryonic endoderm formation and spermatogenic processes (20). In addition, miR-205 has been identified as a regulator of multiple key molecular targets that are essential for modulating cellular proliferation, differentiation, and migratory processes (21).

### 2.2 miR-205’s dual role in cancer

**Figure 2:**
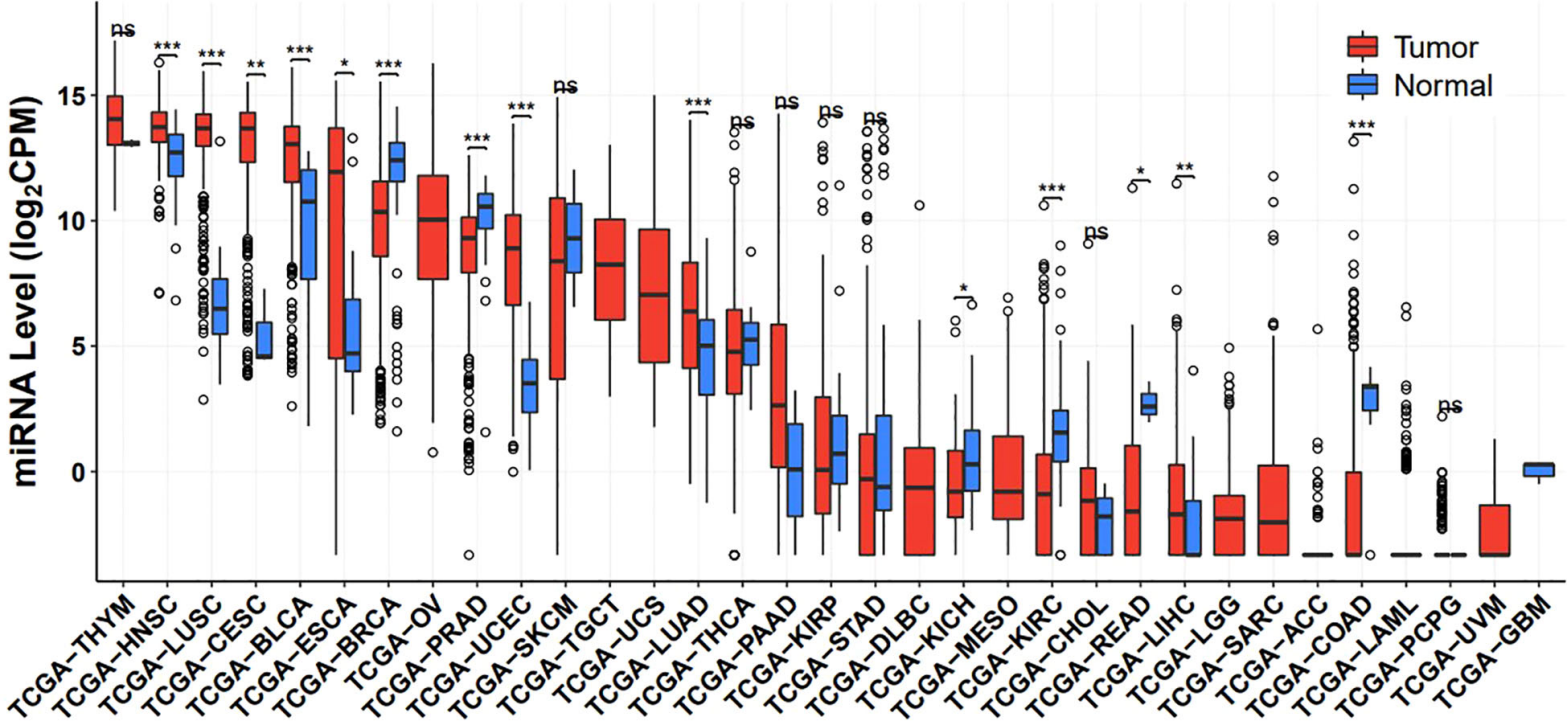
miR-205 expression in The Cancer Genome Atlas (TCGA) pan-cancer (http://bioinfo.jialab-ucr.org/CancerMIRNome/).

Bioinformatics analysis utilizing the TCGA database has revealed significant differential expression patterns of miR-205 across various cancer types compared to adjacent normal tissues (see **Figure 2**). miR-205 is significantly upregulated in multiple malignancies, including non-small cell lung cancer, bladder carcinoma, esophageal adenocarcinoma, ovarian carcinoma, nasopharyngeal carcinoma, endometrioid adenocarcinoma, and head and neck squamous cell carcinomas, where it functions as an oncogenic driver promoting tumorigenic processes. In contrast, miR-205 expression is markedly downregulated in other cancer types, such as breast carcinoma, renal cell carcinoma, prostate adenocarcinoma, and cutaneous melanoma, where it appears to exert tumor-suppressive functions (8, 12). Even in the same cancer, the role of miR-205 may vary depending on the subtype. For example, in triple-negative breast cancer, miR-205 usually plays a tumor suppressor role, whereas in hormone receptor-positive breast cancer, its role may be more complex (22). In the molecular context, the role of miR-205 is highly dependent on the expression patterns of its target genes and regulated signaling pathways in different cancers. For example, in breast cancer, miR-205 inhibits EMT by specifically targeting ZEB1 and ZEB2, thereby exerting tumor suppressor effects (18); Whereas, in cervical cancer cells, up-regulation of miR-205 can significantly target and inhibit CHN1 expression levels, thereby promoting tumor cell proliferation (23). In addition, miR-205 may also regulate tumors by modulating the tumor microenvironment. For example, miR-205 was significantly induced by hypoxia in cervical and lung cancer cells, while significant suppression of ASPP2 was observed. It was confirmed by further studies that the hypoxia-induced ASPP2 inhibition was mainly attributed to miR-205 elevated (24). miR-205 may also influence tumor progression by regulating immune cell functions (immune microenvironment) such as T cells and macrophages. For example, Fan et al. found in non-small cell lung cancer (NSCLC) patients undergoing radiotherapy that radiotherapy upregulated the expression level of miR-205, which promotes autophagy in lung cancer cells, maintains the survival of memory T-cells, and promotes the self-renewal of B1 cells, which facilitates the death of tumor cells and enhances the patient’s anti-tumor immunity (25). In addition, miR-205 expression may also be tightly influenced by epigenetic regulation. miR-205 expression is regulated by DNA methylation and histone modifications. For example, miR-205 is often simultaneously silenced and acquires DNA hypermethylation in muscle-invasive bladder tumors and low-differentiated bladder cell lines, regulating bladder cancer cell proliferation and differentiation (26). Similarly, Kim ES et al. showed that ionizing radiation (IR) enhances the hypermethylation of miR-205-5p CpG islands through activation of Src in lung or breast cancers, leading to a decrease in miR-205-5p expression, which in turn stimulates Bcl-w, mediated proliferation and metastasis of human lung or breast cancer cells (27).

In summary, the dual role of miR-205 may depend specifically on various factors such as cancer type, molecular background and tumor microenvironment. In-depth exploration of whether miR-205 acts as an angel or a devil in different cancers is of great significance for the future research of miR-205.

## 3 miR-205 regulates the mechanism of apoptosis in tumor cells

Apoptosis represents a highly regulated and genetically programmed cellular process that mediates controlled cell death under both physiological homeostasis and pathological conditions (28). It is a key mechanism for maintaining normal cellular homeostasis by removing senescent and diseased cells, thereby supporting overall organismal health and normal cellular functions (29, 30). When cells become cancerous, they acquire the ability to evade apoptosis, which promotes their uncontrolled development. This ability to evade apoptosis has now become a recognized “hallmark of cancer” (2).

Apoptosis is predominantly mediated through two well-characterized molecular pathways: the intrinsic pathway and the extrinsic pathway. The intrinsic pathway, alternatively referred to as the mitochondrial-mediated pathway, is initiated by intracellular stimuli including but not limited to growth factor deprivation, genotoxic stress, and endoplasmic reticulum stress (31). The critical event in the intrinsic apoptotic pathway is mitochondrial outer membrane permeabilization (MOMP). Extensive research has established that BCL-2 family proteins, comprising both pro-apoptotic and anti-apoptotic members, play a crucial regulatory role in tumor progression through their modulation of the mitochondrial apoptotic pathway. This regulatory mechanism is mediated primarily through the controlled release of cytochrome c and subsequent activation of caspase cascades (32). In contrast, the extrinsic apoptotic pathway is initiated through specific ligand-receptor interactions, where death ligands bind to their cognate transmembrane death receptors, triggering downstream apoptotic signaling cascades (33). Dysregulation of death receptor-mediated signaling in the extrinsic apoptotic pathway contributes to tumorigenesis by promoting malignant cell survival and proliferation (34). Both apoptotic pathways converge on the activation of caspase cascades, which execute the biochemical and morphological changes characteristic of programmed cell death (35). Based on these findings, it is hypothesized that the regulation of apoptotic processes may hold significant potential for the development of novel therapeutic strategies in cancer treatment. Analysis of the molecular mechanisms by which miR-205 regulates tumors reveals that it can inhibit cancer progression by controlling the apoptotic processes in tumor cells.

### 3.1 Regulation of endogenous apoptotic pathways by miR-205

Accumulating evidence from numerous studies has demonstrated that miR-205 can directly modulate BCL-2 expression, thereby regulating apoptotic processes in multiple cancer types. Specifically, in prostate cancer cells, laryngeal squamous cell carcinoma (LSCC), and adrenocortical carcinoma (ACC) SW-13 cells, miR-205 upregulation has been shown to significantly reduce both BCL-2 mRNA and protein expression levels. This downregulation subsequently enhances apoptotic activity and inhibits cancer cell proliferation and metastatic potential (36–38). Furthermore, deoxyelephantopin (DET), a bioactive sesquiterpene lactone isolated from Elephantopus scaber (Asteraceae), has been extensively characterized for its potent anti-inflammatory and anti-neoplastic properties. This phytochemical compound has emerged as a promising therapeutic candidate for the treatment of various pathological conditions, particularly due to its demonstrated capacity to inhibit proliferation across multiple cancer cell lines. Notably, a seminal study conducted by Ji et al. revealed that DET induces miR-205 upregulation, which subsequently targets and downregulates BCL-2 expression in colorectal carcinoma cells. This molecular mechanism promotes apoptotic cell death, suggesting DET’s potential as a novel therapeutic agent for clinical oncology applications (39). Qiu et al. demonstrated in their investigation of breast cancer MCF-7 cells that miR-205 overexpression significantly enhances cleaved Caspase-3 expression while concurrently reducing the BCL-2/Bax ratio, thereby inducing apoptotic cell death in malignant cells (40).

Notably, Myeloid cell leukemia-1 (MCL-1), a pro-survival member of the BCL-2 protein family, serves as a critical anti-apoptotic regulator in cellular homeostasis (41). In nasopharyngeal carcinoma cells, transfection with miR-205-5p mimics significantly upregulated the protein expression of both BCL-2 and MCL-1, while concurrently downregulating the expression of pro-apoptotic proteins Bax and Bak. Apoptotic activity is significantly suppressed in nasopharyngeal carcinoma cells (42). ZAROGOULIDIS P et al. demonstrated that miR-205 overexpression in lung adenocarcinoma cell lines A549 and H1975 significantly inhibits Caspase-3 activation and Bax expression, while concurrently upregulating MCL-1 and Survivin protein levels, ultimately resulting in the suppression of apoptotic pathways in malignant pulmonary cells (43).

In summary, miR-205 directly targets and modulates the expression of the anti-apoptotic protein BCL-2 in multiple cancer types, thereby regulating intrinsic apoptotic pathways (in **Table 1**).

**Table 1:** miR-205 regulates tumor cell apoptosis.

Types	Molecular target	Tumor type	References
Endogenous apoptotic			
suppressor	BCL-2↓	Prostate cancer	(36)
	BCL-2↓	LSCC	(37)
	BCL-2↓	ACC	(38)
	BCL-2↓	Colorectal cancer	(39)
	BCL-2↓	Breast cancer	(40)
oncogene	BCL2↑,MCL-1↑,Bax↓,BaK↓	Nasopharyngeal cancer	(42)
	MCL-1↑, Survivin↑, Caspase-3↓, Bax↓	Lung cancer	(43)
Exogenous apoptotic			
suppressor	TNFAIP8↓,P53↑	Thyroid cancer	(46)
	TNFAIP8↓	Lymphoma	(47)
	TRAF2↓, NF-κB↓	Breast cancer	(51)

### 3.2 Regulation of exogenous apoptotic pathways by miR-205

In tumor cells, miR-205 primarily modulates extrinsic apoptosis through the TNFR1/TNF receptor signaling pathway. TNF-α, a transmembrane protein, activates the NF-κB pathway upon binding to its receptor TNF-R1, thereby inducing the expression of TIPE family proteins, including TNFAIP8 and TIPE2 (44). Tumor necrosis factor alpha-induced protein 8 (TNFAIP8) functions as an anti-apoptotic regulator in malignant cell (45). Yang et al. demonstrated that miR-205 overexpression in thyroid carcinoma cells specifically downregulates TNFAIP8 expression while upregulating the pro-apoptotic protein p53, ultimately promoting apoptotic cell death in thyroid cancer cells (46). Similarly, Li et al. demonstrated that miR-205 upregulation in lymphoma cells specifically targets and downregulates TNFAIP8 expression, consequently inducing apoptotic cell death in conjunctival mucosa-associated lymphoid tissue (MALT) lymphoma (47). The TNF-R1 receptor itself possesses a death domain that, under certain conditions, can activate caspase cascades and induce apoptosis (48). Tumor necrosis factor receptor-associated factor-2 (TRAF2), which normally acts in concert with other members of the TRAF protein family, is involved in inhibiting the activation of the NF-κB signaling pathway and stimulating the TNFR response to various mitogen-activated protein (MAP) kinase cascades (49). TRAF2 is established as a critical regulatory component in the activation of the NF-κB signaling pathway (50). In breast carcinoma cells, miR-205 upregulation specifically targets TRAF2, leading to significant downregulation of both TRAF2 mRNA and protein expression levels. This suppression of TRAF2 subsequently inhibits NF-κB signaling pathway activation, ultimately resulting in the attenuation of apoptotic processes (51).

### 3.3 Regulation of other apoptotic factors

#### 3.3.1 PTEN/PI3K-Akt pathway

miR-205 has been demonstrated to regulate the expression of multiple oncogenes and tumor suppressor genes, including but not limited to ZEB1, PTEN, ErbB3, and VEGF-A, through target gene modulation (21), which regulates the PI3K-AKT signaling pathway, thereby mediating the occurrence of cellular apoptosis. In cancer, the PI3K-Akt signaling pathway is often found to be over-activated. This over-activation results in increased cell proliferation, a reduction in apoptosis, and a greater propensity for tumor formation and metastasis (52).

ErbB3, a member of the epidermal growth factor receptor (EGFR) family, functions as a potent oncogenic driver when overexpressed or genetically altered, activating downstream tumorigenic signaling cascades (53). Li and colleagues demonstrated that miR-205 upregulation in prostate carcinoma cell lines specifically targets and suppresses ErbB3 expression, consequently attenuating PI3K-Akt signaling pathway activity. This molecular inhibition results in significant downregulation of BCL-2 expression, concomitant upregulation of Bax and cleaved caspase-3/caspase-9 levels, and ultimately enhances apoptotic cell death in malignant prostate cells (54). VEGF-A, a critical regulator of angiogenesis, plays a pivotal role in promoting tumor-associated vascularization and interacts with PI3K to activate Akt signaling, consequently suppressing apoptotic processes in malignant cells (55, 56). In renal carcinoma cells, miR-205 overexpression results in the significant downregulation of both VEGF-A and PTEN expression. This molecular suppression inhibits PI3K-Akt signaling pathway activation and subsequently induces apoptotic cell death (57, 58). P-glycoprotein (P-gp) represents a membrane-associated drug efflux transporter that is critically involved in mediating multidrug resistance (MDR) phenotypes in cancer cells (59). Li et al. demonstrated that miR-205 overexpression in doxorubicin-resistant hepatocellular carcinoma cells significantly upregulated the expression of its target gene PTEN. This molecular alteration inhibited PI3K-AKT signaling pathway activity, leading to subsequent downregulation of P-gp expression. Consequently, these molecular changes restored chemosensitivity to doxorubicin and induced apoptotic cell death in the previously resistant malignant cells (60).

In lung, ovarian, gastric, and nasopharyngeal carcinomas, miR-205 functions as an oncogenic regulator. Comprehensive statistical analyses demonstrate that in lung cancer (LC) tissues, miR-205 expression is significantly upregulated, while PTEN expression is concurrently downregulated. miR-205-mediated suppression of PTEN results in the marked upregulation of PI3K and phosphorylated AKT (p-AKT) expression levels, ultimately leading to the attenuation of apoptotic processes in malignant pulmonary cells (61). miR-205 overexpression in ovarian or gastric carcinoma cells specifically targets and downregulates PTEN expression, leading to the subsequent upregulation of phosphorylated AKT (p-AKT) levels and ultimately resulting in the suppression of apoptotic cell death (62, 63). Mao et al. demonstrated that transfection with miR-205 mimics in nasopharyngeal carcinoma CNE2 cells significantly upregulated AKT expression while concurrently downregulating PTEN levels, ultimately resulting in the attenuation of apoptotic processes in malignant nasopharyngeal cells (64). Xin et al. identified that the long non-coding RNA LA16c-313D11.11 directly interacts with and suppresses miR-205 activity in endometrial carcinoma, consequently upregulating its target gene PTEN. This molecular interaction indirectly inhibits PI3K-AKT signaling pathway activation and promotes apoptotic cell death in malignant endometrial cells (65).

#### 3.3.2 Other genes

miR-205 has been demonstrated to directly modulate tumor cell apoptosis through the regulation of specific target genes, as comprehensively summarized in **Table 2**. A particularly significant target is protein kinase C epsilon (PRKCE), a member of the protein kinase C (PKC) family, which has been strongly correlated with unfavorable clinical outcomes in gallbladder carcinoma (GBC) (66). Recent studies have established that PRKCE functions as an anti-apoptotic regulator, inhibiting programmed cell death in malignant cells and consequently promoting tumor progression (67). Zhang et al. have elucidated that the overexpression of miR-205 specifically targets and downregulates PRKCE and BCL-2 expression, while simultaneously upregulating pro-apoptotic markers Bax and cleaved caspase-3. This molecular mechanism significantly enhances the induction of apoptosis in gallbladder carcinoma cells (68). Furthermore, in the context of acute lymphoblastic leukemia (ALL), miR-205 targets the PTK7 gene. Overexpression of miR-205 results in decreased levels of PTK7 at both the protein and mRNA levels, significantly increasing the apoptosis rate of ALL cells (69). YAP1 is usually considered as a carcinogen in tumors, which can promote the development of many cancers, including gastric cancer (70). Xian et al. have demonstrated that miR-205 overexpression specifically targets and downregulates YAP1 expression in gastric cancer cell lines SGC-7901 and HGC-27. This regulatory mechanism leads to a significant alteration in the expression of apoptosis-related proteins, characterized by decreased BCL-2 levels and concomitant upregulation of both caspase-3 and BAX, ultimately promoting programmed cell death in gastric carcinoma cells (71). Studies have shown that circRNAs can be used as a microRNA sponge to chelate microRNA, thus affecting the expression of target mRNA and dynamically regulating the process of mRNA translation (72). Xu et al. demonstrated that hsa_circ_0001429 downregulates miR-205 expression in breast carcinoma through molecular sequestration, consequently upregulating its target gene KDM4A and ultimately suppressing apoptotic processes in malignant breast cells (73). Similarly, circ_NOTCH3 interacts with miR-205 and targets KLF12, leading to the downregulation of KLF12 expression in basal-like breast cancer cells. This molecular interaction promotes tumor progression and suppresses apoptotic processes (74).

**Table 2:** miR-205 targets other genes to regulate tumor cell apoptosis.

Types	Molecular target	Tumor type	references
suppressor	PRKCE↓, Bax↑, cleaved caspase-3↑,BCL-2↓	Gallbladder cancer	(68)
	PTK7↓	ALL	(69)
	YAP1↓, BCL-2↓, cleaved caspase-3↑, Bax↑	Gastric cancer	(70)
	KDM4A↓	Breast cancer	(71)
	KLF12↑	Breast cancer	(72)
oncogene	CHN1↑	Cervical cancer	(23)
	AXLN2↓, Wnt/β-catenin↓	Cervical cancer	(75)
	YAP1↓	Colon cancer	(76)

CHN1, a GTPase-activating protein, has been identified as a potential target of miR-205 through bioinformatics analysis. Liu et al. demonstrated that CHN1 mRNA expression is significantly elevated in cervical carcinoma tissues compared to adjacent non-neoplastic tissues. Downregulation of miR-205 directly targets and upregulates CHN1 expression, consequently suppressing apoptotic processes in cervical cancer cells (23). Niu et al. further demonstrated that miR-205 is regulated by the long non-coding RNA HNRNPU-AS1 in cervical cancer. They observed that elevated HNRNPU-AS1 levels inhibit miR-205 expression, leading to the upregulation of its target gene AXIN2 and subsequent activation of the Wnt/β-catenin signaling pathway. This signaling cascade is known to promote apoptotic cell death and suppress cervical cancer progression (75). Similarly, in colorectal carcinoma, miR-205 exhibits oncogenic properties. Jin et al. demonstrated that the long non-coding RNA ZEB1-AS1 directly targets and downregulates miR-205 in colon cancer cells. This molecular interaction results in the upregulation of YAP1 expression and a subsequent increase in apoptotic cell death in malignant colorectal cells (76).

**Figure 3:**
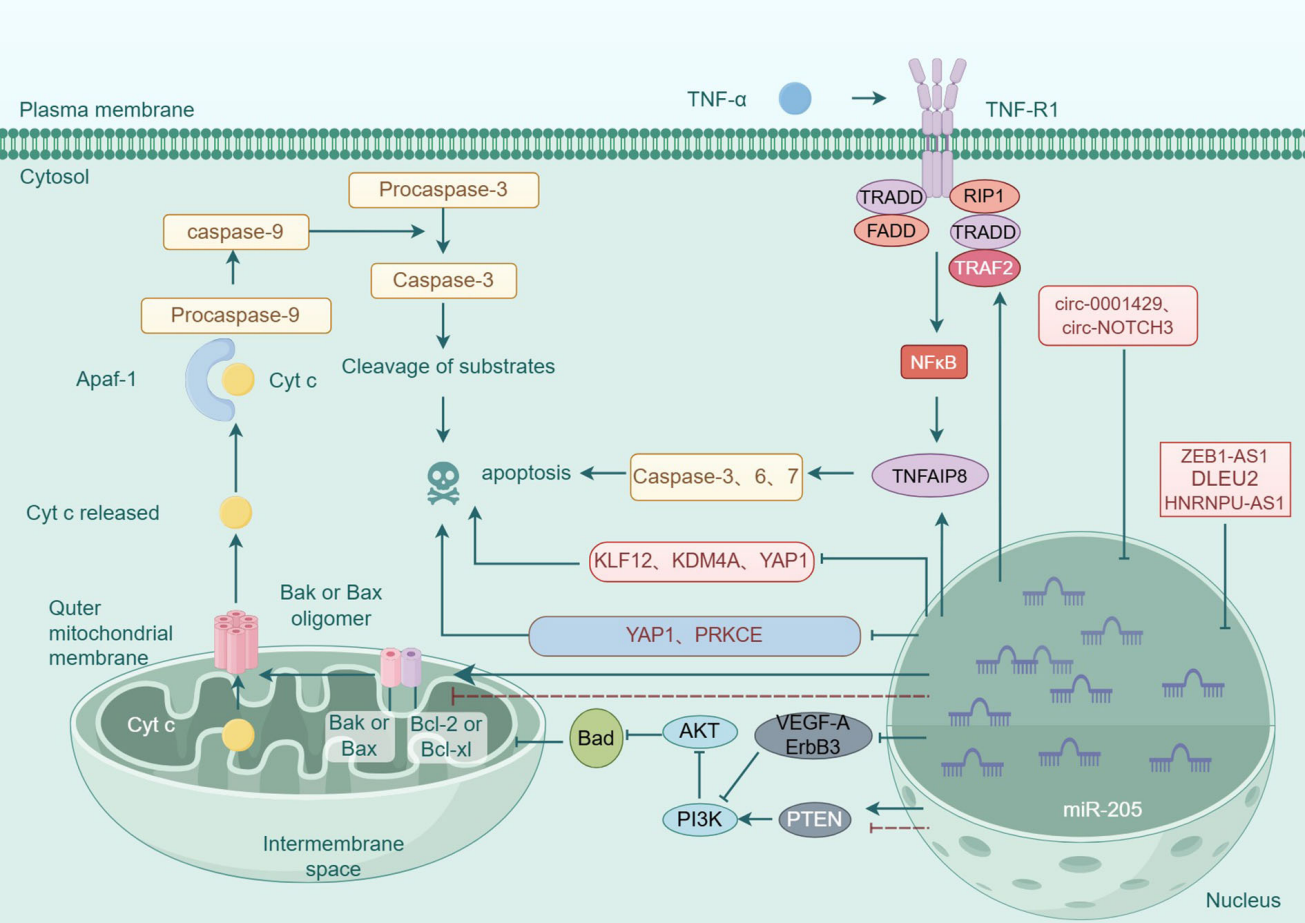
Molecular mechanism of miR-205 regulation of apoptosis in tumor cells.

**Figure 4:**
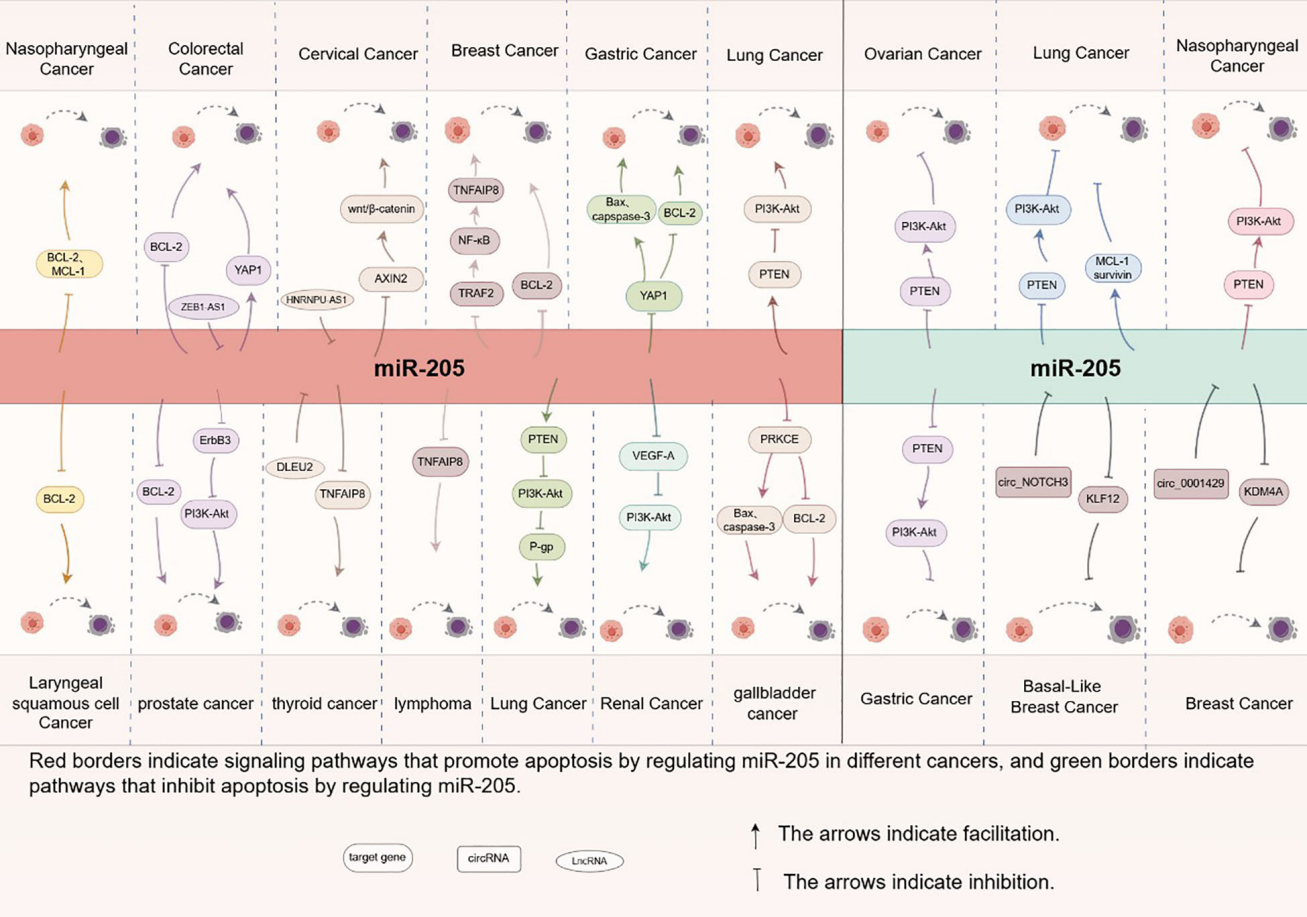
Molecular mechanism of apoptosis regulation by miR-205 in different tumor cells.

In summary, miR-205 plays a critical role in regulating cellular apoptosis through the modulation of multiple genes and signaling pathways (see **Figure 3**), exhibiting both oncogenic and tumor-suppressive functions depending on the cellular context (see **Figure 4**). Recent studies have identified novel apoptosis-related signaling pathways, including those mediated by endoplasmic reticulum stress. However, research specifically investigating miR-205’s role in apoptotic regulation remains in its early stages and requires further experimental validation. These findings highlight the necessity for more comprehensive studies to elucidate the precise molecular mechanisms and functional impacts of miR-205.These molecular insights highlight miR-205’s therapeutic potential, as discussed below.

## 4 The value in cancer treatment

Current therapeutic strategies for cancer, including radiotherapy and chemotherapy, primarily exert their anti-tumor effects by inducing DNA damage to trigger tumor cell death. However, malignant cells with impaired apoptotic pathways frequently develop resistance to these conventional treatments (77). Therefore, exploring novel therapeutic strategies to induce tumor cell apoptosis represents a critical approach for modulating specific molecular pathways and advancing cancer treatment (in **Table 3**).

**Table 3:** therapeutic role of miR-205.

Therapeutic strategy	Molecular target	Tumor type		References
medication				
DET	BCL-2	Colorectal cancer	stress generation, NF-κb inhibition, mitochondrial dysfunction	(39)
Trastuzumab	BCL-2↓	Breast cancer	reducing cell proliferation, blocking cell cycle	(82)
Nano therapy				
MNP Nanopreparations	——	prostatic cancer	reducing cell proliferation, blocking cell cycle reversed drug resistance, inhibited cell proliferation, migration, invasion and induced apoptosis	(8)
gold nanoparticles	PKCϵ	prostatic cancer	Inhibit cell proliferation	(91)
Micelles	ZEB1, cadherin	E-pancreatic cancer	Inhibit cell proliferation, invasion and migration	(94)

### 4.1 Additive effects with drugs

Pharmacological treatment remains a cornerstone of cancer therapy, as numerous traditional medicinal compounds have demonstrated the capacity to inhibit tumor migration and invasion while inducing apoptosis, thereby suppressing cancer progression. To date, various bioactive compounds derived from traditional medicines have shown significant efficacy in clinical applications. DET, a sesquiterpene lactone isolated from the Compositae plant Elephantopus scaber L., promotes apoptosis in hepatocellular carcinoma (HCC) cells through mechanisms involving oxidative stress generation, NF-κB inhibition, and mitochondrial dysfunction (78, 79). Ji et al. demonstrated that DET upregulates miR-205 expression in colon cancer cells, thereby promoting apoptotic cell death. Mechanistically, DET enhances miR-205 expression in malignant colon cells and significantly increases chemosensitivity through the miR-205/BCL-2 signaling axis, resulting in potent antitumor effects (39). Trastuzumab is a chemotherapeutic agent currently widely used for the treatment of HER2-positive breast cancer (80). In clinical trials, Whittle JR et al. confirmed the downregulated expression of miR-205 in a patient-derived xenograft model obtained from trastuzumab-resistant tumors (81). In order to utilize miR-205 as a therapeutic tool in the treatment of breast cancer, Piovan C et al. observed that higher miR-205 expression was significantly associated with a better prognosis by analyzing 52 patients with HER2+ BC who were clinically treated with adjuvant trastuzumab. In addition, their study demonstrated that restoring miR-205 to reverse trastuzumab resistance could further improve the therapeutic efficacy of trastuzumab by reducing cell proliferation and blocking cell cycle progression (82).

### 4.2 Role in drug resistance of cancer cells

Many studies have shown that aberrant expression of miRNAs may be associated with resistance to anticancer drugs. Resistance mechanisms are often associated with changes in related proteins such as PTEN, PDCD4, P-gp and MDR1. In turn, changes in proteins may be directly related to mutations, aberrant expression or translocation of miRNA coding genes, which may affect the expression of related miRNAs, leading to alterations in the function of the target mRNAs, thereby affecting the expression of the target proteins, and thus silencing the target genes fundamentally (83). The 3 ‘ UTR of mRNAs contains binding sites for important translational regulatory elements, including miRNAs, cytoplasmic polyadenylation elements (CPEs), proteins and protein complexes. Deletions in the 3’ UTR of the target mRNA also lead to deletion of the miRNA binding site, which results in loss of miRNA function. to et al. demonstrated that in drug-resistant S1MI80 cells, there was an approximately 1500-bp deletion in the 3 ‘ UTR of the downstream target gene of ABCG2 mRNA of hsa- miR-519c, and thus the miRNA was unable to bind to the ABCG2 mRNA binding, resulting in ABCG2 overexpression in drug-resistant tumor cells (84). In addition, drug transport is an important part of drug disposition. p-glycoprotein, multidrug resistance-associated protein (MRP) and breast cancer resistance protein (BCRP) are closely related to multidrug resistance. Breast cancer resistance protein (BCRP/ABCG2) is a molecular determinant of the pharmacokinetic properties of many human drugs. pan et al. found that miR-328 negatively regulated BCRP expression, and inhibition of miR-328 led to an increase in BCRP protein levels in MCF/MX100 cells, which enhanced drug efflux, decreased cellular drug concentration, and ultimately led to the drug resistance phenotype (85). Currently, most studies on miRNAs related to cellular drug resistance have focused on apoptosis and drug transporters. Once one of the molecules involved in apoptosis is altered, a drug resistance phenotype may emerge. miRNA down- or up-regulation affects the expression of drug transporters, drug targets, or apoptosis- and cell cycle-related components, and thus affects cellular drug resistance (86). For example, Bhatnagar N et al. found that up-regulation of miR-205 and miR-31 down-regulated the downstream target gene Bcl-w and promoted apoptosis levels, restoring the sensitivity of prostate cancer cells to chemotherapy (87).

Therefore, miRNAs are expected to serve as biomarkers of chemotherapy resistance.

### 4.3 miR-205-based nanotherapies

There are two main types of miRNA delivery vectors, viral and non-viral delivery systems. Viral vectors are commonly used for efficient transfer of various genes, oligonucleotides, siRNAs and miRNAs into various target cells or tissues/organs. Several viral vectors such as adenoviral, retroviral and lentiviral vectors have been used for preclinical and clinical evaluation. All of these vectors are very effective in achieving higher delivery efficiencies, however, their poor loading capacity, high toxicity levels and immunogenicity induction limit their clinical translation (88, 89). Therefore, the development of non-viral vectors has received much attention due to the successful and stable delivery of miRNAs.

Nanotechnology-based delivery is a potential method for safely delivering miRNAs and overcoming these associated barriers. Nanotherapies were initially designed primarily to deliver anticancer drugs. However, it has since been discovered that nanoparticles can also successfully deliver nucleic acid molecules such as DNA, RNA, and proteins/antibodies (90). Chauhan N et al. established a preparation technique/methodology for the successful generation of MNP nanopreparations. It was also demonstrated that the prepared MNP nano-formulations containing miR-205 were safe for use in cellular systems. In two prostate cancer cell lines, C4–2 and PC-3, this preparation achieved excellent cellular internalization by endocytosis, escaping endosomal and lysosomal degradation. In addition, they combined this novel MNP miR-205 formulation with docetaxel. It was found that upregulation of miR-205 successfully reversed drug resistance and sensitized prostate cancer cells to docetaxel treatment. It also significantly inhibited prostate cancer cell proliferation, migration, invasion and induced apoptosis (8). Another miR-205 nano-formulation based on gold nanoparticles delivers miR-205 to prostate cancer cells. This reduces protein kinase C Epsilon (PKCϵ) levels and inhibits prostate cancer cell proliferation (91). A cationic copolymer formulation (micelles) prepared by Mittal et al. This micellar formulation has higher stability to miR-205 with particle sizes ranging from 62 nm to 122 nm. It was used to deliver miR-205 and gemcitabine in pancreatic cancer to sensitize drug-resistant cells to drug treatment and inhibit cancer cell proliferation. Meanwhile, the expression level of E-cadherin was up-regulated and that of ZEB1 was down-regulated, inhibiting pancreatic cancer cell invasion and migration. In addition, as miR-205 reversed the drug resistance of these cells, *in vivo* results showed that the tumor growth and weight were significantly reduced after treatment with the gemcitabine-miR-205 complex formulation (92).

In summary, nano-formulation-based delivery of miR-205 is expected to improve the targeted efficacy of cancer therapy.

## 5 The role of other regulated cell death mechanisms

In addition to apoptosis, cell death can occur through various other mechanisms, including autophagy, necroptosis, necrosis, and ferroptosis.

Autophagy, a regulated form of programmed cell death, constitutes a cellular adaptive mechanism that relies on lysosomal degradation to respond to adverse environmental stimuli (93). Under normal physiological conditions, autophagy can degrade aging organelles and unwanted protein, and recover its products for energy sources and raw materials for anabolism (94). However, dysregulated or excessive autophagy can induce a distinct form of programmed cell death, known as autophagic cell death (95, 96). Recent studies have shown that miR-205 can directly modulate the expression of specific tumor suppressor genes and autophagy-related factors, thereby regulating autophagic processes in cancer cells (97, 98).

Zhuo et al. discovered that miR-205 is significantly upregulated in endometrial carcinoma (PR) cells, where it suppresses PTEN expression, leading to the activation of the AKT/mTOR signaling pathway. This molecular cascade subsequently enhances the conversion of autophagy marker LC3-I to LC3-II and upregulates Beclin1 protein levels, ultimately promoting autophagic cell death in malignant cells (99). Furthermore, TP53INP1 directly interacts with key autophagy-related molecules, including LC3 and Atg8 family proteins, thereby facilitating autophagic processes (100). Wang et al. demonstrated that miR-205 upregulation directly targets and suppresses TP53INP1 expression, thereby inhibiting radiation-induced autophagic processes in prostate carcinoma cells (98).

It is crucial to recognize that the interplay between autophagy and apoptosis is highly complex. Indeed, these two cellular processes share common regulatory stimuli and signaling pathways, while exhibiting a degree of mutual inhibition under specific conditions (101).

Beclin1, a critical component in autophagosome formation, serves as a direct substrate for caspase-8. The interaction between Beclin1 and caspase-8 has been observed across multiple cell types and plays a regulatory role in both apoptotic and autophagic processes. For instance, studies have demonstrated that caspase-8-mediated downregulation of Beclin1 expression suppresses autophagic activity during herpes simplex virus infection (102). Furthermore, AKT plays a pivotal role in the cross-regulation of apoptotic and autophagic pathways. Diao et al. demonstrated that AKT phosphorylation not only enhances autophagic activity but also downregulates the expression of key apoptosis-related factors, including Bax and caspases (103).

These findings highlight the complex interplay between miR-205, apoptotic pathways, and autophagic processes, establishing a robust foundation aimed at elucidating their molecular interactions.

Furthermore, in recent years, the concept of “PANoptosis” has emerged as a significant research focus. PANoptosis represents a dynamic form of programmed cell death that integrates molecular features of pyroptosis, apoptosis, and necroptosis (104). PANoptosis plays a critical role in the pathogenesis and progression of diverse diseases, including infectious diseases, malignancies, neurodegenerative disorders, and inflammatory conditions (105). Studies have demonstrated that pyroptosis not only suppresses tumor cell proliferation but also establishes a tumor-promoting microenvironment, ultimately facilitating cancer progression (106). Both apoptosis and necroptosis are recognized as essential anti-cancer mechanisms (107). As a higher-order version of these three death pathways, PANoptosis likely plays a crucial role in therapeutic strategies for disease management. Zhang et al. found that upregulating miR-18a expression in MC3T3-E1 cells significantly suppressed the protein levels of hypoxia-inducible factor-1α (HIF1-α) and NLRP3, thereby promoting PANoptosis of osteoblasts in response to TNF-α induction (108). Furthermore, Wang et al. analyzed kidney clear cell carcinoma (ccRCC) samples from The Cancer Genome Atlas (TCGA) database and three Gene Expression Omnibus (GEO) datasets. They identified seven upregulated miRNAs (hsa-miR-155-5p, hsa-miR-15a-5p, hsa-miR-16-5p, hsa-miR-181a-5p, hsa-miR-21-5p, hsa-miR-210-3p, and hsa-miR-223-3p) and two downregulated miRNAs (hsa-miR-141-3p and hsa-miR-200a-5p), which were significantly associated with PANoptosis-related prognostic features. These findings demonstrate that miRNAs are associated with PANoptosis in tumor cells and may represent a novel therapeutic strategy for clear cell renal cell carcinoma (ccRCC) (109). Currently, investigations into the relationship between miRNAs and PANoptosis remain in the preliminary stages. Although the potential link between miR-205 and PANoptosis in tumor cells has not yet been elucidated, it represents a promising area for future research, potentially leading to the development of novel therapeutic strategies for cancer treatment.

## 6 Conclusions

miRNAs have significantly advanced our understanding of diverse biological processes in organisms. miRNAs play pivotal roles in nearly all biological pathways and are intricately linked to tumor development and progression. Consequently, miRNAs have emerged as promising biomarkers and are being actively developed as novel tools for cancer diagnosis, prognosis, and therapeutic intervention. Recent studies have demonstrated that miR-205 regulates the cell cycle, promotes cellular differentiation, induces apoptosis, and modulates tumorigenesis and cancer progression. miR-205 has been identified as a critical biomarker in oncology, underscoring its clinical significance. Additionally, miR-205 serves as a direct target for certain pharmacological agents, offering potential therapeutic benefits. It can also be used as a direct target for certain drugs to treat diseases. Some studies have proved that miR-205 can be used in combination with some chemotherapeutic drugs to play a role in the treatment of cancer. It can also correct the resistance of many drug-resistant cells to drugs, which is of great significance for the clinical treatment of various cancers. Existing evidence indicates that miR-205 exhibits diverse roles across different cancer types, either promoting or inhibiting apoptosis depending on the specific cancer type and cellular context. The complex regulatory functions of miR-205 and its multifaceted biological effects necessitate further investigation. These findings highlight the potential of targeting miR-205 expression as a promising strategy for cancer therapy.

Apoptosis is a fundamental regulatory mechanism essential for maintaining organismal homeostasis and preventing aberrant cell proliferation. Dysregulation of apoptotic pathways contributes to the pathogenesis of various diseases, including cancer. Extensive research has revealed that miR-205 modulates apoptosis-related signaling pathways and targets key genes to either promote or inhibit apoptosis, thereby influencing cancer progression. The ability of miR-205 to induce apoptosis in malignant cells has significant implications for advancing medical and healthcare practices, making it a rational and increasingly utilized therapeutic target. miR-205 has the potential to enhance the efficacy of chemotherapeutic agents; however, comprehensive clinical trials are required to validate its therapeutic potential.

The regulation of apoptosis through miRNA-mediated mechanisms represents a critical approach in cancer treatment. Although significant research has been conducted on the role of miRNAs in apoptosis regulation across various cancers, several unresolved issues remain. For instance: (1) How can drugs that induce apoptosis via miRNA modulation be optimally selected for clinical application? (2) Do these therapeutic agents alter intracellular miRNA levels upon administration, and could they potentially induce adverse effects or secondary diseases? Despite these challenges, miRNA-mediated apoptosis induction holds considerable promise as a future strategy for cancer therapy.

miR-205 plays crucial roles in cancer progression and apoptosis regulation, yet its clinical translation faces significant challenges. First, miR-205 displays distinct functional duality across cancer types: while acting as a tumor suppressor in prostate cancer, it exhibits oncogenic properties in specific lung cancer subtypes. This context-dependent functionality underscores the importance of tumor microenvironment in determining miR-205’s actions, highlighting the need for comprehensive characterization of its tissue-specific regulatory networks to develop precise therapeutic strategies.

The major obstacle in miR-205-based therapy involves delivery system optimization. Two critical issues must be addressed: (1) achieving tumor-specific delivery of miR-205 modulators (mimics or inhibitors), and (2) improving their *in vivo* stability. Future research directions should include: (1) Systematic identification of novel miR-205 targets in emerging cell death pathways (e.g., pyroptosis, cuproptosis) using organoid-AI integration platforms. (2) Comprehensive mapping of miR-205 regulatory networks through single-cell sequencing and CRISPR screening. (3) Development of advanced delivery platforms (e.g., exotic-based or nanomaterial systems) to enhance tumor targeting.

Currently, miR-205 research stands at the critical juncture between basic science and clinical implementation. Interdisciplinary integration of molecular biology, bioinformatics, materials science, and clinical research will be essential to overcome current limitations, ultimately transforming miR-205 into a viable precision oncology tool while advancing our fundamental understanding of cell death regulation.
